# Sparsity Is Better with Stability: Combining Accuracy and Stability for Model Selection in Brain Decoding

**DOI:** 10.3389/fnins.2017.00062

**Published:** 2017-02-17

**Authors:** Luca Baldassarre, Massimiliano Pontil, Janaina Mourão-Miranda

**Affiliations:** ^1^Laboratory for Information and Inference Systems, École Polytechnique Fédérale de Lausanne (EPFL)Lausanne, Switzerland; ^2^Istituto Italiano di TecnologiaGenoa, Italy; ^3^Department of Computer Science, University College LondonLondon, UK; ^4^Max Planck University College London Centre for Computational Psychiatry and Ageing Research, University College LondonLondon, UK

**Keywords:** sparse methods, structured sparsity, model selection, reproducibility, predictive models

## Abstract

Structured sparse methods have received significant attention in neuroimaging. These methods allow the incorporation of domain knowledge through additional spatial and temporal constraints in the predictive model and carry the promise of being more interpretable than non-structured sparse methods, such as LASSO or Elastic Net methods. However, although sparsity has often been advocated as leading to more interpretable models it can also lead to unstable models under subsampling or slight changes of the experimental conditions. In the present work we investigate the impact of using stability/reproducibility as an additional model selection criterion^1^ on several different sparse (and structured sparse) methods that have been recently applied for fMRI brain decoding. We compare three different model selection criteria: (i) classification accuracy alone; (ii) classification accuracy and overlap between the solutions; (iii) classification accuracy and correlation between the solutions. The methods we consider include LASSO, Elastic Net, Total Variation, sparse Total Variation, Laplacian and Graph Laplacian Elastic Net (GraphNET). Our results show that explicitly accounting for stability/reproducibility during the model optimization can mitigate some of the instability inherent in sparse methods. In particular, using accuracy and overlap between the solutions as a joint optimization criterion can lead to solutions that are more similar in terms of accuracy, sparsity levels and coefficient maps even when different sparsity methods are considered.

## 1. Introduction

Supervised machine learning techniques are being increasingly used in neuroimaging analysis for their inherent ability to deal with multivariate data, higher sensibility and possibility of incorporating specific prior-information.

Given the high-dimensionality of neuroimaging, and the few number of samples, regularized linear models have been applied in order to produce effective predictive models (Mourao-Miranda et al., 2006, 2007; Grosenick et al., 2011; Michel et al., 2011). However, ordinary linear models, such as, the Least Squares Ridge Regression (Tikhonov and Arsenin, 1977) or standard Support Vector Machines (SVMs) (Cortes and Vapnik, 1995) employ an l2 regularization scheme, hence they are incapable of discriminating which areas (or voxels) of the brain mostly contribute to the model's predictions. In other words, these models are dense, in the sense that they use the information contained in the entire voxel set to generate a predictive function.

Sparse methods, like the LASSO (Tibshirani, 1996) or the Elastic Net (Zou and Hastie, 2005), are able to estimate solutions for which only few voxels are deemed relevant, therefore aiding interpretation. However, often these models provide overly sparse solutions, where the non-zero coefficients are assigned to disparate regions across the brain, without exploiting any spatial or temporal prior information (Grosenick et al., 2011; Michel et al., 2011; Rasmussen et al., 2012).

Structured sparsity models (Chambolle, 2004; Bach et al., 2011; Baldassarre et al., 2012a; Micchelli et al., 2013) extend the well-known methods of LASSO by encouraging models which are sparse in some preferred way, e.g., the non-zero regression coefficients may be preferred to be associated to the same brain region or nearby voxels. Furthermore, the coefficients may be encouraged to be constant or vary smoothly within regions of the brain. Despite sparsity has traditionally been connected with interpretability, in the sense that sparser models are easier to interpret, these new structured sparsity models promise an even greater ease of interpretation of the coefficient maps, because the active voxels are grouped together in possibly few clusters, which fits well with our knowledge about the brain's specialized regions and networks. These method hence have the potential to further improve out-of-sample performance in comparison to standard sparsity methods such as the LASSO.

### 1.1. Structured sparse models in neuroimaging

Recently, (structured) sparsity methods have received significant attention in neuroimaging, see Gramfort et al. (2013), Mohr et al. (2015), Belilovsky et al. (2015), Jenatton et al. (2012), Hoyos-Idrobo et al. (2015), Dohmatob et al. (2014), and Grosenick et al. (2013) and references therein. For example Jenatton et al. (2011) investigated the benefits of using hierarchical structured sparsity for brain decoding, taking into account the spatial and multi-scale structure of the fMRI data. Their proposed approach yielded similar or higher prediction accuracy than the compared approaches (l1 and squared l2 regularization penalties), and the obtained map of weights or coefficients exhibited a cluster-like structure. Fiot et al. (2014) compared a number of structured sparse methods (Sobolev, total variation, fused LASSO) with regularization methods which do no take into account the spatial structure (LASSO, Ridge and Elastic-Net) on a clinical classification problem. Their results showed that the structured sparse approaches can lead to coherent and improved coefficient maps with better classification performance than the ones obtained with the standard regularization methods.

Mohr et al. (2015) presented a comparison of different sparse and non sparse regularization methods for brain decoding. They focused on a classification problem and use the Logistic Loss or Hinge Loss (SVMs). The authors argued that l1 regularization can improve classification performance over l2 approaches (using SVM as an example of an l2 approach) as well as improve model interpretability. In addition, by considering the 3D structure of fMRI data, even better interpretability of the weights or coefficient maps could be possible. For this purpose, one more promising method which was not considered in Mohr et al. (2015) is sparse total variation. This method has been suggested in the context of fMRI by Baldassarre et al. (2012b) as means to learn interpretable and more stable brain maps. Further work investigating applications of sparse total variation in this context include Gramfort et al. (2013), Dohmatob et al. (2014), and Eickenberg et al. (2015).

Despite of all the evidence that sparsity and structured sparsity can lead to predictive models that are easier to interpret, sparsity alone is not sufficient for making reasonable inferences as sparse models can be unstable under subsampling or slight changes of the experimental conditions. One key source of instability is correlation between features, a problem specific to multivariate methods but not univariate methods. However, univariate methods are often too simplistic and may be suboptimal. Another difficulty with sparse models is that there are many possible ways of imposing sparsity or structured sparsity in predictive models. Finding the ideal sparsity for a specific problem is therefore a model selection problem. A common difficulty in neuroimaging applications is that often different models lead to very similar generalization performance (e.g., accuracy), then it becomes difficult to choose the best model and identify the “true brain map” of informative or predictive regions. Some authors have used the capacity to recover the “best brain regions” as alternative criterion to evaluate the models. In theses cases the “best regions” are based either on prior knowledge about the problem or univariate statistical tests applied to the data, both of which might not correspond to the ground truth. In fact, in most neuroimaging applications we do not know a-priori which regions are expected to be relevant for prediction therefore alternative approaches for model comparison are necessary.

One way to increase the stability or reproducibility of sparse models is to explicitly account for it during the model selection procedure. The use of a tradeoff between accuracy and reproducibility as a model selection criterion has been previously proposed in neuroimaging (e.g., Strother et al., 2002; Rasmussen et al., 2012). For example, in Rasmussen et al. (2012), the authors investigated the relative influence of model regularization parameter choices on both the model generalization and the reliability of the spatial patterns (coefficient maps) extracted from a classification model. Building upon their work, we advocate stability/reproducibility as the natural counterpart of sparsity in order to obtain interpretable inferences from sparse supervised learning methods.

The issue of improving interpretability and stability of predictive brain maps has also been studied from a different perspective by Hoyos-Idrobo et al. (2015) and Wang et al. (2014). In Hoyos-Idrobo et al. (2015) the authors focused on feature clustering and bagging as a means to improve stability of l1 regularization and interpretability of the associated brain maps. In Wang et al. (2014) the authors proposed a “randomized structural sparsity,” incorporating the idea of structural sparsity in the stability selection framework. They demonstrated that their proposed approach can achieve better control of false positives and false negatives than alternative methods.

### 1.2. Our contribution

In this paper, we investigate the role of model selection criteria on different sparsity (and structured sparsity) methods that have been recently applied for decoding fMRI data, including one we proposed in a previous work (Baldassarre et al., 2012b), and assess their performance with respect to accuracy, sparsity and reproducibility. In order to investigate the impact of using reproducibility as an additional criterion for model selection, we compare three different model selection criteria: (i) classification accuracy alone; (ii) classification accuracy and overlap between the solutions (or coefficient maps); (iii) classification accuracy and correlation between the solutions (or coefficient maps). The methods we consider include LASSO (Tibshirani, 1996), Elastic Net (Zou and Hastie, 2005), Total Variation (Michel et al., 2011), Graph Laplacian Elastic Net (GraphNET) (Grosenick et al., 2011) and sparse Total Variation (Baldassarre et al., 2012b). For our comparison, we use a dataset of fMRI scans collected from 16 healthy volunteers while watching pleasant or unpleasant images (Mourao-Miranda et al., 2006, 2007; Hardoon et al., 2007).

Model selection is performed by a Leave-One-Subject-Out Cross-Validation (LOSO-CV) scheme, which we describe in detail in the methods section. Although regularization helps to reduce model variance, the value of the regularization parameter(s) which yield a maximal accuracy model varies across the cross-validation folds, resulting in models with varying degree of sparsity and sets of selected variables (voxels). A main point of this paper is to show that this instability effect can be substantially reduced by employing a different model selection criterion which involves accuracy and “reproducibility” simultaneously. Specifically, we discuss the relevance of our findings with respect to using classification accuracy as a proxy for statistical significance of a given model. Our results suggest that the model selection criterion plays a more important role than the choice of the sparsity or structured sparsity. When using sparsity and overlap between the solutions as a joint optimization criterion the solutions for different methods became very similar in terms of accuracy, sparsity levels and coefficient maps. These results demonstrate the added value of accounting for reproducibility/stability in addition to generalization performance during model selection in supervising learning models.

The paper is organized in the following manner. In Section 2 we present the sparse (and structured) methods, experimental protocol, model selection criteria and dataset. We present the results in Section 3 and the discussion in Section 4.

## 2. Materials and methods

### 2.1. Supervised learning for classification

Given a training set of input-output pairs $D={\left\{\left(x_{i},y_{i}\right)\right\}}_{i=1}^{m}$, with $x_{i}\in {\mathbb{R}}^{p}$ and *y*_*i*_ ∈ ℝ, a supervised learning method infers the relationship between *x* and *y* by estimating a function *f* : ℝ^*p*^ → ℝ such that, for every *x* ∈ ℝ^*p*^, *f*(*x*) provides the prediction of *y* given *x*.

In neuroimaging studies, the input *x*_*i*_ represents the brain scans in vector format and the number of variables *p* corresponds to the number of recorded voxels. In the present paper we consider a binary classification task, so that *y* ∈ {−1, 1}, but our results can easily be extended to the regression or the multi-class setting. Furthermore, we limit our analysis to linear models, so that the decision function can be written as *f*(*x*) = sign(*x*^*T*^β), where β ∈ ℝ^*p*^ is a vector of coefficients to be estimated, one associated to each voxel.

The aim of a machine learning algorithm is to find a coefficient vector β able to classify new examples and with specific properties such as sparsity (i.e., few non-zero coefficients) or smoothness. Regularization methods find β by minimizing an objective function consisting of a data fit term *E*(β) and a penalty term Ω(β) that favors certain properties and improves the generalization over unseen examples (outside the training set D).

As data fitting term we consider the square loss that can be concisely written as

$$E\left(\beta \right)=\frac{1}{m}||X\beta -Y|{|}_{2}^{2}$$

where *X* ∈ ℝ^*m*×*p*^ is the matrix that contains the training examples as rows and $Y={\left(y_{1},\ldots ,y_{m}\right)}^{T}$ is the column vector formed by the target variables.

### 2.2. Structured sparsity models

Note that, since for a linear model each regression coefficient is associated to a voxel, the vector β can also be interpreted as 3D matrix of the same size as the brain scans and we use this 3D structure to define particular penalty functions β ↦ Ω(β). We define the ℓ_1_ norm of β as $\left||\beta |{|}_{1}=\overset{p}{\underset{i=1}{\sum }}|\beta_{i}\right|$; the discrete gradient of β in 3 dimensions as ∇β, with

$$\begin{array}{l} {\left(\nabla \beta \right)}_{i,j,k}^{1}=\beta \left(i,j,k\right)-\beta \left(i-1,j,k\right)\\ {\left(\nabla \beta \right)}_{i,j,k}^{2}=\beta \left(i,j,k\right)-\beta \left(i,j-1,k\right)\\ {\left(\nabla \beta \right)}_{i,j,k}^{3}=\beta \left(i,j,k\right)-\beta \left(i,j,k-1\right) \end{array}$$

and ${\left(\nabla \beta \right)}_{i,j,k}^{\ell }=0$ if (*i, j, k*) is on the boundary w.r.t. the direction ℓ. Finally, $\underset{i\sim j}{\sum }{\left(\beta_{i}-\beta_{j}\right)}^{2}$ means that the sum is only for neighboring voxels *i* and *j*.

For each method, the model $\hat{\beta }$ is estimated by solving the optimization problem

(1) $$\underset{\beta \in R^{p}}{\min}\left\{E(\beta )+\Omega (\beta )\right\}$$

where Ω(β) is defined as follows.

#### 2.2.1. Elastic net (ENET) and LASSO

$$\Omega \left(\beta \right):=\lambda_{1}||\beta |{|}_{1}+\lambda_{2}||\beta |{|}_{2}^{2}.$$

This regularizer entails a tradeoff between variable selection and coefficient shrinkage. For λ_2_ = 0, we obtain the LASSO, while λ_2_ ≠ 0 allows for correlated features to be selected together. Notice also that unlike the structured sparsity regularizers described below, the location of the non-zero components is not constrained in any manner.

#### 2.2.2. Total variation (TV)

$$\Omega \left(\beta \right):=\lambda ||\nabla \beta |{|}_{1}.$$

This regularizer favors solutions that have constant value in contiguous regions and has its origins in image de-noising applications (Rudin et al., 1992), however it does not enforce any coefficient to be exactly zero.

#### 2.2.3. Sparse total variation (STV)

$$\Omega \left(\beta \right):=\lambda \left(||\nabla \beta |{|}_{1}+||\beta |{|}_{1}\right).$$

By adding a ℓ_1_-penalty term to the Total Variation functional, this regularize favors solutions whose coefficients are constant within contiguous regions, but also promotes sparsity. This hybrid method has been proposed in other domains, such as image de-noising using Fourier or wavelet representations (see e.g., Ma et al., 2008) and was applied to brain decoding in our previous work (Baldassarre et al., 2012b). Notice also that (Sparse) Total Variation reduces to a fused Lasso in 3D space (Dohmatob et al., 2014).

#### 2.2.4. Laplacian (LAP)

$$\Omega \left(\beta \right):=\frac{1}{2}\lambda \underset{i\sim j}{\sum }{\left(\beta_{i}-\beta_{j}\right)}^{2}.$$

This regularizer relaxes the constancy requirement of the Total Variation method, allowing for smooth variations within regions. It is called “Laplacian” since the regularizer can be rewritten as $\overset{p}{\underset{i,j=1}{\sum }}\beta_{i}\beta_{j}L_{ij}$, where the matrix *L* is the Laplacian associated to a 3D grid graph modeling neighboring voxels.

#### 2.2.5. Sparse Laplacian (SLAP)

$$\Omega \left(\beta \right):=\frac{1}{2}\lambda \left(1-\alpha \right)\underset{i\sim j}{\sum }{\left(\beta_{i}-\beta_{j}\right)}^{2}+\lambda \alpha ||\beta |{|}_{1}$$

where α ∈ [0, 1]. This regularizer encourages both smooth variations within regions and sparsity of the regression vector. The corresponding method is similar to GraphNET (Grosenick et al., 2011), with λ_1_ = λα and λ_*G*_ = λ(1 − α). Note also that LAP corresponds to the special case that α = 0. In all cases, λ and α are hyper-parameters (often referred to as regularization parameters) that control the trade-off between the data fitting term, typically measured by classification accuracy, and the degree of regularization, which measures the parsimony (sparsity) of the model. These hyper-parameters must be chosen in an unbiased way during learning. The numerical algorithm employed to solve the optimization problem (Equation 1) is outlined in Appendix 1 (Supplementary Material).

### 2.3. Experimental protocol and assessment

In this section we present details about the experimental protocol used, including criteria for model selection and measures used to assess the performance of the different methods.

Our aim is to provide a consistent and unbiased procedure in order to best compare different supervised learning methods that goes beyond the simple prediction accuracy performance measure. For this purpose, we introduce two measures of model reproducibility/stability and study their impact for model selection and model assessment.

#### 2.3.1. Nested cross-validation

We perform two nested loops of Leave-One-Subject-Out Cross-Validation (LOSO-CV). The external loop is used for assessing the classification accuracy, the sparsity and the stability of the methods; the internal loop is used for selecting the hyper-parameter(s) in each method (e.g., λ_1_ and λ_2_ for Elastic Net). Hence, for each method, we train *N* different models, where *N* is the number of subjects in the dataset. Note that each subject has many examples of each class, therefore the LOSO-CV used in the present work does not correspond to the commonly used Leave-One-Out Cross-Validation (LOO-CV) procedure, where only one example is left for test in each cross-validation fold.

#### 2.3.2. Thresholding

Although the sparse methods should yield sparse coefficient vectors, due to numerical approximations during optimization some of the estimated coefficients might not have been set exactly to zero. Therefore, we adopt the heuristic of setting to zero the smallest components of the regression vector which contribute to only 0.01% to the ||β||_1_. Specifically we reorder the components of β so that |β_1_| ≥ |β_2_| ≥ ⋯ ≥ |β_*p*_|, choose the smallest integer *r* such $\overset{r}{\underset{k=1}{\sum }}|\beta_{k}|\geq \left(1-10^{-4}\right)||\beta |{|}_{1}$ and set to zero the components β_*r*+1_, …, β_*p*_.

#### 2.3.3. Performance measures

Let β(*s*) (the *signature*) be the coefficient vector estimated when the data for subject *s* is left out for testing. We define the model or signature support *I*_*s*_: = {*i* | β(*s*)_*i*_ ≠ 0} the index set of the locations of the non-zero coefficients (or sparsity pattern), the model *sparsity* $S\left(s\right):=\frac{\left|I_{s}\right|}{p}$ as the relative number of non-zero coefficients and the *pairwise relative overlap* as

$$O_{s,s\prime }:=\frac{\left|I_{s}\cap I_{s\prime }\right|}{\max\left(\left|I_{s}|,|I_{s}^{\prime }\right|\right)}.$$

We then define the *corrected pairwise relative overlap* as

$$O_{s,s\prime }^{c}:=\frac{|I_{s}\cap I_{s\prime }|-E}{\max\left(\left|I_{s}|,|I_{s}^{\prime }\right|\right)},$$

where *E* is the expected overlap between the support of two random vectors with sparsity *S*(*s*) and *S*(*s*′), respectively, given by the formula^2^

(2) $$\begin{array}{l} E=pS\left(s\right)S\left(s\prime \right). \end{array}$$

The correction term *E* compensates the fact that the pairwise relative overlap increases with the size of the sparsity pattern of the models, see Rasmussen et al. (2012) for a discussion; note also that in that paper model sparsity is defined as the number of non-zero coefficients.

Next we define the **average pairwise overlap**

(3) $$\begin{array}{l} \bar{O}:=\frac{1}{N\left(N-1\right)}\overset{N}{\underset{s\neq s\prime =1}{\sum }}O_{s,s\prime } \end{array}$$

and the **average corrected pairwise overlap**

(4) $$\begin{array}{l} {\bar{O}}^{c}:=\frac{1}{N\left(N-1\right)}\overset{N}{\underset{s\neq s\prime =1}{\sum }}O_{s,s\prime }^{c} \end{array}$$

as measures of *stability* and *corrected stability*, respectively.

As a further measure of stability we also use the **average pairwise correlation** defined as

(5) $$\begin{array}{l} \bar{C}:=\frac{1}{N\left(N-1\right)}\overset{N}{\underset{s\neq s\prime =1}{\sum }}C_{s,s\prime }, \end{array}$$

where *C*(*s, s*′) is the sample Pearson's correlation between β(*s*) and β(*s*′).

The **accuracy** of a method is the average percentage of correctly classified examples over all the LOSO folds, namely

(6) $$\begin{array}{l} \text{Accuracy}=\frac{1}{N}\overset{N}{\underset{s=1}{\sum }}\frac{1}{m_{s}}\overset{m_{s}}{\underset{i=1}{\sum }}\delta \left(f_{s}\left(x_{i}\right)=y_{i}\right) \end{array}$$

where $f_{s}\left(x_{i}\right)=\text{sign}\left(\beta {\left(s\right)}^{T}x_{i}\right)$ and *m*_*s*_ is the number of examples for subject *s*.

### 2.4. Model selection

Since we are interested in evaluating methods not only according to classification accuracy, but also with respect to measures of reproducibility/stability such as correlation and corrected overlap, it behooves us to consider these measures also during model selection. Obviously, the stability measures by themselves cannot directly be used for model selection, because selecting the model's hyper-parameters that only maximize stability will yield highly biased models that do not actually learn from data. For instance, a model with just one constant non-zero coefficient will maximize both correlation and corrected overlap, but won't be able to accurately predict.

**Figure 1:**
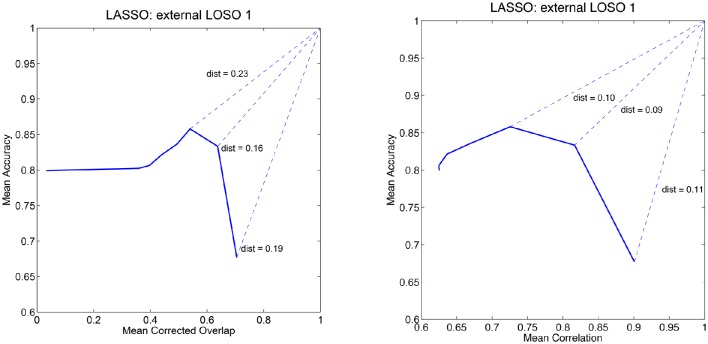
**Mean accuracy vs. mean corrected overlap (left)** and mean accuracy vs. mean correlation **(right)** for the first external LOSO fold for the LASSO model. The curves are obtained by varying the regularization parameter and computing the measures across the internal LOSO folds.

Henceforth, adopting and extending the procedure proposed by Rasmussen et al. (2012), we consider both prediction accuracy and either correlation or corrected overlap simultaneously^3^. We can use a diagram to visualize the dependency between accuracy and one of the stability measures: by varying the model's hyper-parameters values we obtain different points on this diagram. Ideally, we would like to find the values that yield exactly the point (1, 1), that is perfect accuracy and perfect stability. However, since this hardly happens in practice, we are satisfied with the hyper-parameters values that yield the point closest (with respect to the Euclidean distance) to (1, 1) in either the accuracy vs. correlation or accuracy vs. corrected overlap diagrams. An example of these diagram is reported in Figure 1 for the LASSO.

Summarizing, we consider three model selection criteria:

1. **Accuracy-based**. The model hyper-parameters are selected to maximize the classification accuracy over the internal LOSO-CV.
2. **Corrected overlap based**. The hyper-parameters are selected in order to minimize the distance to (1, 1) in the mean accuracy vs. mean corrected overlap diagram. Note that this criteria was only applied to the sparse methods (LASSO, ENET, STV, and SLAP).
3. **Correlation-based**. The hyper-parameters are selected in order to minimize the distance to (1, 1) in the mean accuracy vs. mean correlation diagram.

### 2.5. Multi-measure assessment

**Figure 2:**
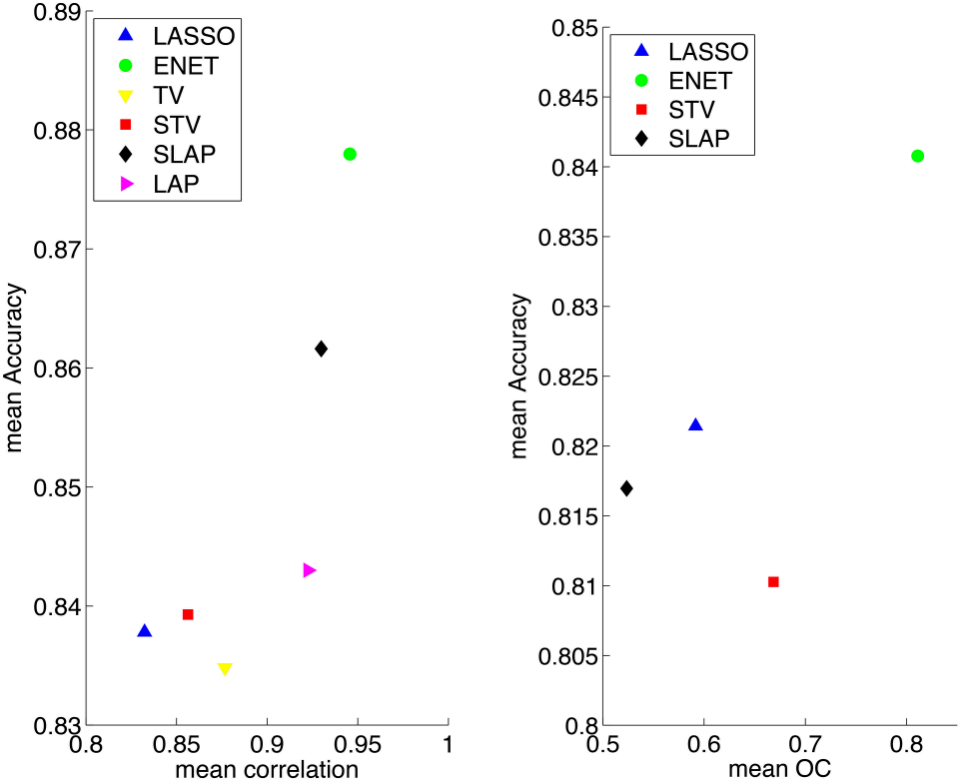
**Summary results for different models when the model selection criteria Acc/Corr and Acc/OC are employed**.

The accuracy vs. stability diagrams introduced in the previous section for model selection can also be used for model assessment. In fact, when training a model and selecting its hyper-parameters in order to maximize both accuracy and stability, it is appropriate to compare its performance to other methods with respect to the same criterion used for model selection. In this case, each method yields a single point in the accuracy vs. stability diagram and we can both visually assess the differences in the methods—which is the most accurate, which is the most stable and which obtains the best balance between accuracy and stability—but also quantitatively compute their distances to the ideal (1, 1) point. In Figure 2 we use these diagrams to visualize the performance of the various methods on the dataset considered in this paper.

### 2.6. Dataset

We used fMRI data from 16 male healthy US college students (age 20–25) (Mourao-Miranda et al., 2006, 2007; Hardoon et al., 2007). Participants did not have any history of neurological or psychiatric illness, had normal vision and had given written informed consent to participate in the study after the study was explained to them.

The fMRI data were acquired on a 3T Allegra Head-only MRI system, using a T2^*^ sequence with 43 axial slices (slice thickness, 3 mm; gap between slices, 0 mm; *TR* = 3 s; *TE* = 30 ms; *FA* = 80°; FOV = 192 × 192 mm; matrix, 64 × 64; voxel dimensions, 3 × 3 × 3 mm).

#### 2.6.1. fMRI experimental design

There were three different active conditions: viewing unpleasant (dermatological diseases), neutral (people) and pleasant images (pretty girls in swimsuits), and a control condition (fixation). In each run, there were 6 blocks of the active condition (each consisting of 7 brain scans) alternating with control blocks (fixation) of 7 brain scans. The six blocks of each of the 3 stimuli were presented in random order. Here we focus the analyses on two active conditions: viewing unpleasant and pleasant images. The fMRI scans acquired during the pleasant and unpleasant conditions (considering an hemodynamic delay of 3 s) defined the input patterns.

#### 2.6.2. Preprocessing

The data were pre-processed using SPM2^4^. All the scans were realigned to remove residual motion effects and transformed into standard space (Talairach and Tournoux, 1988). The data were de-trended and smoothed in space using an 8 mm Gaussian filter. Finally, a mask was applied to select voxels that have probability 0.5 or higher of being located in gray matter. This operation nearly halves the number of voxels from 219, 727 to 122, 128. The preprocessed dataset consists of 1344 scans of size 122, 128 voxels, with 42 scans per subject per active condition.

### 2.7. Summarization of the coefficient maps

In order to summarize the coefficient maps for the sparse methods (LASSO, ENET, STV and SLAP) we listed the clusters according to their extension using the script 3dclust from AFNI (https://afni.nimh.nih.gov/afni/) and found the brain regions correspondent to the maximum coefficient within each cluster using the software Talairach Daemon (http://www.talairach.org/daemon.html).

## 3. Results

In this section, we describe the experimental results obtained applying the different sparsity regularization methods as well as non sparse ones to decode the mental state (i.e., viewing pleasant or unpleasant images) of the subject left out of the LOSO-CV as described in the methods section. The Matlab code one may use to reproduce our results is available at https://github.com/lucabaldassarre/neurosparse.

The main aim of the experiments is to compare three different model selection criteria: (i) classification accuracy, (ii) classification accuracy and overlap between the solutions, (iii) classification accuracy and correlation between the solutions; and investigate their impact on the different performance measures: *Accuracy, Sparsity, Correlation, Overlap*, and *Corrected Overlap (OC)*. As we noted in the previous section, the last three quantities are stability/reproducibility measures which indicate the extent to which maps of coefficients or sparsity patterns associated with a learning method are stable across LOSO-CV folds and hence reproducible.

Table 1 reports the average and standard deviation for the five performance measures computed on the external LOSO-CV (test error) of each learning method and model selection criterion. Each row in the table refers to one learning method trained with one of the three model selection criteria: “Acc,” “Acc/OC,” and “Acc/Corr.” For example, in the table,“LASSO - Acc” means that we run LASSO and selected its regularization parameter according to best accuracy, whereas “LASSO - Acc/OC” means that we run LASSO and selected the regularization parameter which minimizes the distance in the Accuracy/Corrected Overlap diagram. Likewise, “LASSO - Acc/Corr” means that we run LASSO and selected the regularization parameter which minimizes the distance in the Accuracy/Correlation diagram. As expected when test performance is measured according to accuracy, the most effective model selection criterion is accuracy itself. Similarly, when test performance accounts for correlation or corrected overlap, the best performance tends to be obtained by using Acc/Correlation or Acc/OC as the model selection criterion, respectively. In Figure 1, we show an example of “Mean Accuracy vs. Mean Corrected Overlap” and of “Mean Accuracy vs. Mean Correlation” plot for the LASSO model trained for the first external LOSO fold. Note that for each learning method, there is significant discrepancy between the different optimization criteria used.

**Table 1:** **Model performance**.

**Method**	**Accuracy**	**Correlation**	**Sparsity**	**Overlap**	**OC**
LASSO - Acc	86.31 ± 5.86%	69.63 ± 11.92%	0.64 ± 0.11%	53 ± 12%	52 ± 12%
LASSO - Acc/OC	82.12 ± 8.42%	76.13 ± 19.30%	0.25 ± 0.07%	59 ± 20%	59 ± 20%
LASSO - Acc/Corr	83.78 ± 5.53%	83.24 ± 2.49%	0.27 ± 0.03%	67 ± 4%	66 ± 4%
E-Net - Acc	88.02 ± 5.51%	92.06 ± 7.93%	84.14 ± 21.27%	77 ± 24%	2 ± 3%
E-Net - Acc/OC	84.08 ± 6.39%	96.00 ± 0.82%	3.00 ± 0.09%	84 ± 2%	81 ± 2%
E-Net - Acc/Corr	87.80 ± 5.42%	94.57 ± 1.56%	88.39 ± 14.07%	83 ± 14%	2 ± 4%
TV - Acc	85.79 ± 5.10%	86.86 ± 6.99%	n.a.	n.a.	n.a.
TV - Acc/Corr	83.48 ± 6.69%	87.68 ± 8.23%	n.a.	n.a.	n.a.
STV - Acc	85.86 ± 5.30%	52.40 ± 17.72%	12.37 ± 14.38%	25 ± 19%	20 ± 16%
STV - Acc/OC	81.03 ± 7.15%	86.23 ± 15.07%	2.56 ± 0.86%	69 ± 22%	67 ± 22%
STV - Acc/Corr	83.93 ± 4.70%	85.63 ± 8.71%	39.97 ± 24.93%	47 ± 25%	21 ± 17%
SLAP - Acc	87.05 ± 5.93%	72.66 ± 18.04%	10.77 ± 6.50%	42 ± 31%	35 ± 25%
SLAP - Acc/OC	81.70 ± 6.80%	80.66 ± 16.07%	1.18 ± 1.28%	53 ± 33%	52 ± 32%
SLAP - Acc/Corr	86.18 ± 5.63%	92.98 ± 1.29%	3.34 ± 0.09%	78 ± 2%	75 ± 2%
Lap - Acc	83.71 ± 5.30%	85.51 ± 7.86%	n.a.	n.a.	n.a.
Lap - Acc/Corr	84.97 ± 5.67%	91.72 ± 7.39%	n.a.	n.a.	n.a.

One interesting observation from Table 1 is the fact that using accuracy and stability (measured by OC) as a joint criterion for model selection leads to solutions that are more similar across different sparsity models in terms of accuracy, sparsity levels and corrected overlap, with respect to using only accuracy as optimization criterion. For example, when using the criterion Acc/OC the accuracy across different sparsity models varies from 81.03% (STV) to 84.08% (E-NET), the sparsity varies from 0.25 (LASSO) to 3 (E-NET) and the corrected overlap (OC) varies from 52 (SLAP) to 82 (E-NET). These variations are much smaller than the ones observed for model selection based on accuracy only. In this case the accuracy varies from 83.71% (LAP) to 88.02% (E-NET), the sparsity varies from 0.64 (LASSO) to 84.14 (E-NET) and the corrected overlap (OC) varies from 2 (E-NET) to 35 (SLAP). Interestingly, when using the criterion Acc/Corr this effect is not observed.

In Figure 2, we present the results for the different methods in the planes “Mean Accuracy vs. Mean Corrected Overlap” and “Mean Accuracy vs. Mean Correlation.” Overall the best performing method is the Elastic Net, achieving an average accuracy of over 84% when the model selection criterion Acc/OC is employed, yielding at the same time a highly stable sparsity pattern. The LASSO gives the most sparse coefficient vectors (maps), however these are less stable than those obtained by Elastic Net.

**Figure 3:**
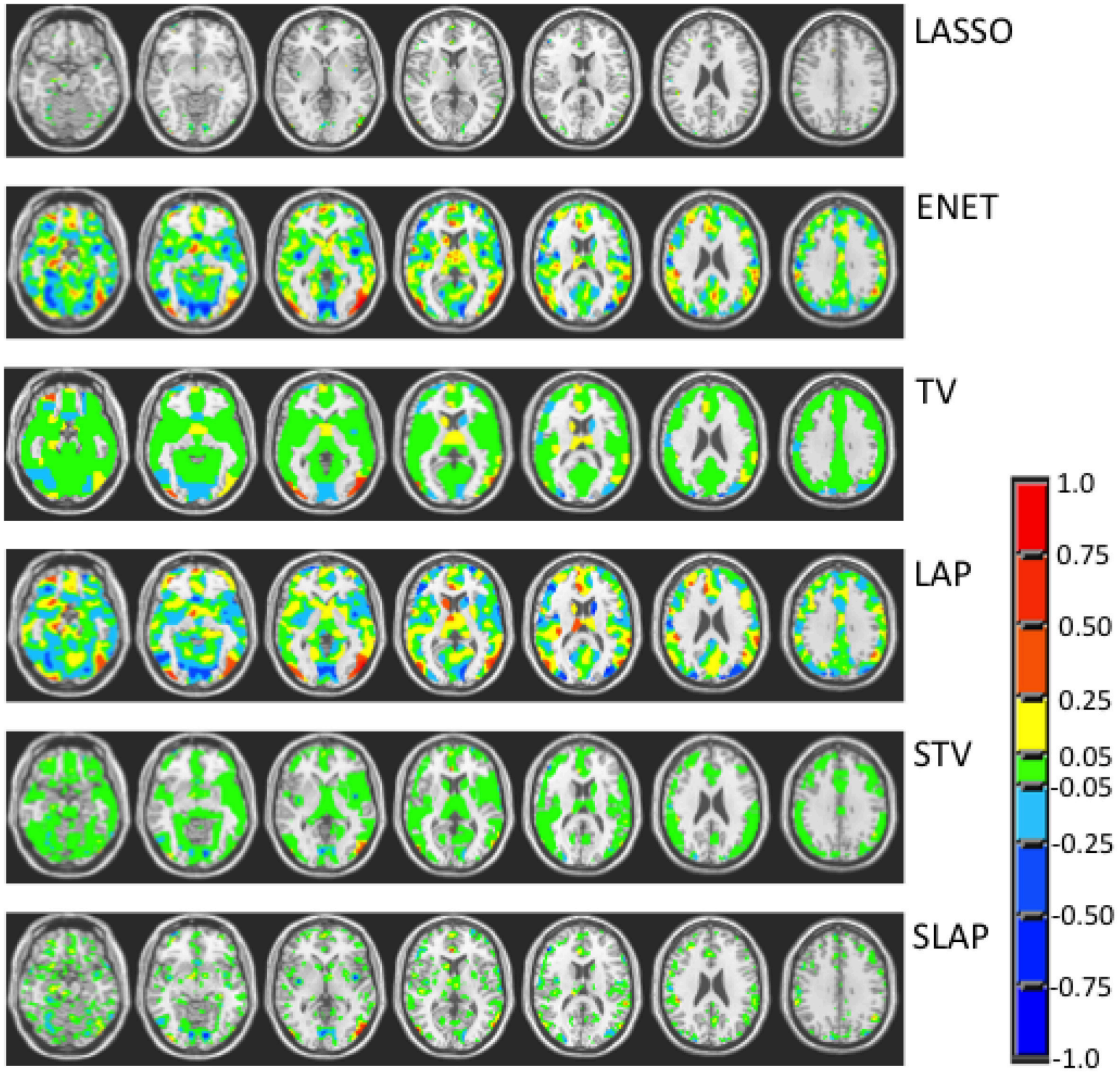
**Coefficient maps for different models when the model selection criterion Acc is employed**.

**Figure 4:**
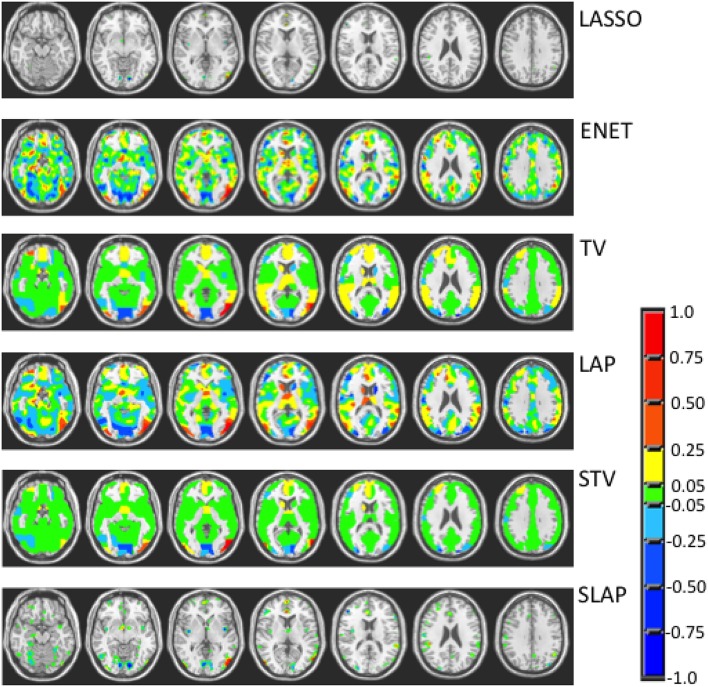
**Coefficient maps for different models when the model selection criterion ACC/Corr is employed**.

**Figure 5:**
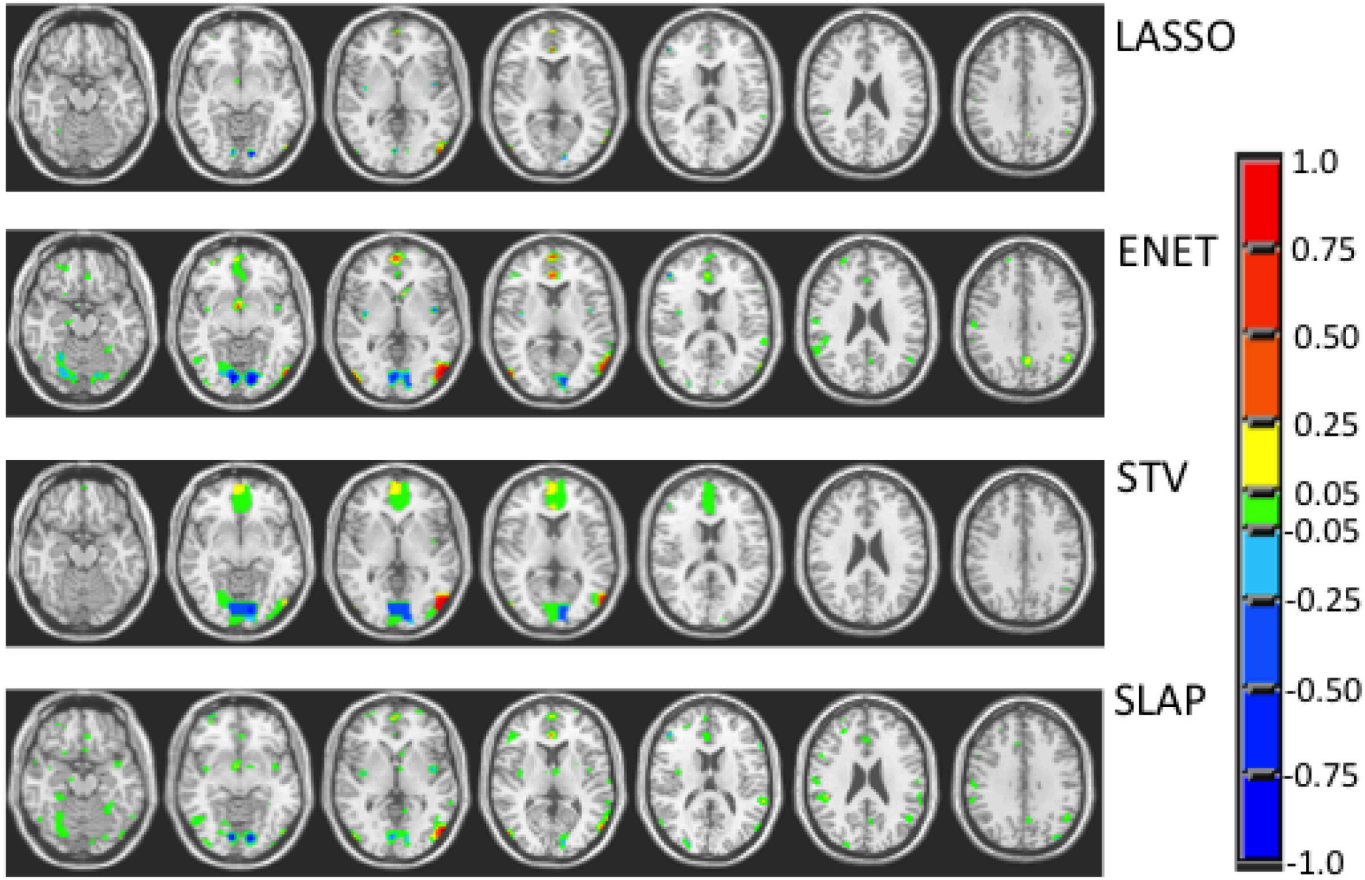
**Coefficient maps for different sparse models when the model selection criterion Acc/OC is employed**.

Figures 3–**5** show the coefficient maps for the different methods and different optimization criteria (averaged across the external LOSO folds). Figure 3 shows the coefficient maps when accuracy was employed as a model selection criterion. It is possible to notice that the coefficient maps present very different levels of sparsity and smoothness even though the accuracy for the different methods varies less than 5% (from 83.71 to 88.02%). These results illustrate the effect of different sparsity constraints on the coefficient maps. As expected the LASSO solution is extremely sparse regardless of the model selection criterion. The coefficient maps for ENET are not sparse and show a smooth variation over the voxels/regions. Not surprisingly, the coefficient maps for Total Variation (TV) and Laplacian (LAP) methods led to non-sparse solutions, however the TV coefficients show large regions with constant values while the LAP coefficients show a smooth variation across the brain voxels/regions, presenting a similar pattern as the ENET. Finally, the coefficient maps for Sparse Total Variation (STV) and Sparse Laplacian (SLAP) are sparser versions of the TV and LAP. Including correlation across the LOSO solutions as an additional model selection criterion leads to coefficient maps similar to the ones obtained using only accuracy (Figure 4), with STV showing a pattern similar to TV.

In Figure 5, we can see the coefficient maps when accuracy and corrected overlap (OC) were employed as model selection criteria for the sparse methods (LASSO, ENET, STV, and SLAP). It is interesting to see that in this case all maps became very similar both in terms of sparsity and smoothness. In all cases the coefficient maps are very sparse with well localized clusters and peaks. Overall, the Acc/OC model selection criterion seems to lead to solutions that are the most sparse and stable (according to the overlap and corrected overlap measures), while the observed decrease in accuracy performance is not significant (according to the Welch's *t*-test).

**Figure 6:**
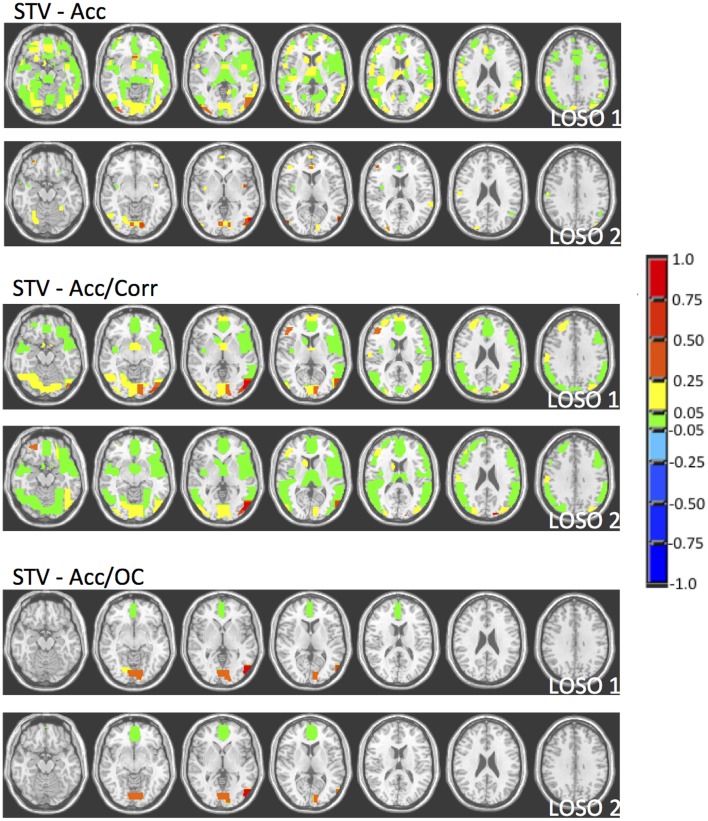
**Coefficient maps for the first two LOSO folds for the STV method using different model selection criteria (Accuracy, Accuracy and Correlation, Accuracy and Corrected Overlap)**.

In order to illustrate the impact of the model selection criteria on the stability of the coefficients across folds, in Figure 6 we present the coefficient maps for the first and second LOSO folds for the STV method using the different model section criteria. As we can see there is a lot of variation between the coefficients when the regularization parameters are selected according to accuracy, whereas when distance in the “Mean Accuracy vs. Mean Corrected Overlap” diagram is used the coefficients become very similar which indicates that the solutions become more reproducible/stable.

A more objective description of the coefficient maps is provided in Tables 2–4 with a list of the five top clusters found by each method and each model selection criterion. This description was not done for the non sparse approaches as in this case all voxels within the image are included in a single cluster. The Tables show the total number of clusters found and the brain regions (including the corresponding Brodman Area - BA) corresponding to the five top clusters ranked according to the maximum coefficient within the cluster. It is possible to see that although different methods find very different numbers of clusters depending on the sparsity constraint and model selection criteria, there is a good agreement between the main clusters found by the different methods. In particular when the optimization criteria Acc/OC was used the different sparse methods (LASSO, ENET, STV, and SLAP) identified the same regions (Table 4), with some differences in the ranking order. As expected, considering the fMRI experimental task (visualization of pleasant or unpleasant pictures), the main clusters include visual areas (e.g., left and right middle occipital gyrus), areas often associated with emotional processing (e.g., right medial frontal gyrus, right anterior cingulate) and cerebellum (left cummen). A visual inspection of the non-sparse approaches shows that their peaks are also in similar regions selected by the sparse approaches.

**Table 2:** **List of clusters for sparse methods when the model selection criterion Acc is employed**.

**Method**	**Number of clusters**	**Top five clusters**
LASSO	61	Left middle occipital gyrus - BA 19
		Right middle frontal gyrus - BA 11
		Right middle occipital gyrus - BA 19
		Left culmen
		Left superior temporal gyrus - BA22
STV	35	Right middle occipital gyrus - BA19
		Left superior frontal gyrus - BA 8
		Left inferior parietal lobule - BA 40
		Right inferior temporal gyrus - BA 20
		Right inferior frontal gyrus - BA 47
SLAP	49	Left middle occipital gyrus - BA 37
		Right middle occipital gyrus - BA19
		Right middle frontal gyrus - BA 11
		Right postcentral gyrus - BA 7
		Right anterior cingulate - BA 24

**Table 3:** **List of clusters for sparse methods when the model selection criterion Acc/Corr is employed**.

**Method**	**Number of clusters**	**Top five clusters**
LASSO	60	Left middle occipital gyrus - BA 37
		Right middle occipital gyrus - BA19
		Left culmen
		Right middle frontal gyrus - BA 11
		Right anterior cingulate - BA 24
SLAP	148	Left middle occipital gyrus - BA 37
		Right middle occipital gyrus - BA19
		Right postcentral gyrus - BA 7
		Right anterior cingulate - BA 24
		Left culmen

*In this case we only present the list of cluster for LASSO and SLAP since the other approaches did not lead to sparse solutions*.

**Table 4:** **List of clusters for sparse methods when the model selection criterion Acc/OC is employed**.

**Method**	**Number of clusters**	**Top five clusters**
LASSO	62	Left middle occipital gyrus - BA 37
		Right middle occipital gyrus - BA 19
		Left culmen
		Right middle frontal gyrus - BA 11
		Right anterior cingulate - BA 24
ENET	71	Left middle occipital gyrus - BA 37
		Right middle occipital gyrus - BA19
		Right medial frontal gyrus - BA10
		Right anterior cingulate - BA 24
		Left culmen
STV	10	Left middle occipital gyrus - BA 37
		Right middle occipital gyrus - BA19
		Right anterior cingulate - BA 24
		Right middle frontal gyrus - BA 11
		Left culmen
SLAP	106	Left middle occipital gyrus - BA 37
		Right middle occipital gyrus - BA19
		Right medial frontal gyrus - BA10
		Right anterior cingulate - BA 24
		Left culmen

## 4. Discussion

During the last years there has been a huge increase in the application of machine learning methods to analyse neuroimaging data (see Pereira et al., 2009; Haynes, 2015, and references therein), varying from neuroscience applications for decoding mental or cognitive states (e.g., Polyn et al., 2005; Mourao-Miranda et al., 2006; Haynes et al., 2007; Schrouff et al., 2012) to clinical applications for diagnoses and prognoses (e.g., Kloppel et al., 2012). Despite their inherent ability to deal with multivariate data and their predictive framework which enables decisions at the single subject level, one of their main limitations is the interpretability issue. For linear machine learning models, the vector of coefficients (also known as weight vector) can be plotted and visualized as a brain image showing the contribution of each voxel in the image to the decision function. The main issue is that, for non sparse approaches (e.g., Kernel Ridge Regression, Support Vector Machines) all voxels within the image will have some contribution to the decision function, making it difficult to decide which voxels contribute the most. A number of approaches have been proposed to ease the interpretability issue, such as feature selection (e.g., Martino et al., 2008; Langs et al., 2011; Rondina et al., 2014), searchlight (Kriegeskorte et al., 2006) and sparse models (see references in the introduction). Sparsity has often been advocated as a proxy for interpretability, however sparsity can be imposed by very different penalties or constraints which should be related to prior knowledge about the problem considered.

Our previous work (Baldassarre et al., 2012b) showed that different sparsity constraints can lead to similar performance in terms of accuracy, but the resulting models differ in term of sparsity and stability. In the present work, we investigated the impact of the model selection criteria (parameter optimization criteria) on models with different sparsity penalties. Our results show that having a second criterion (in addition to accuracy) for model selection improves the stability/reproducibility (measured by OC or correlation across LOSO solutions) of the sparse models, i.e., the instability of the sparse models decreases by including reproducibility as an additional model selection criterion.

When trying to interpret brain maps resulting from sparse models it is important to have in mind that the properties of the maps, such as sparsity and smoothness, are strongly driven by the choice of the penalty term in the objective function. The effect of the chosen penalty on the map of coefficients can be clearly seen in Figure 3. It is possible to observe that when accuracy is used for model selection, methods with different penalties can have similar performance and very different coefficient maps. However, when reproducibility (measured by OC) is used in addition to accuracy for models selection, the solutions or maps became more similar even across the different sparse methods (Figure 5). Interestingly the same effect is not observed when reproducibility (measured by correlation) is used as additional model selection criterion (Figure 4). One possible explanation for the difference observed on the results when using OC or correlation representing stability is the fact that correlation is significantly affected, i.e., reduced, by differences in models' supports. Therefore, correlation between models can be very high when the models are both very sparse or both very dense, leading to choosing such models during model selection, as evidenced by Figure 4. We also noticed that the criteria Acc/OC tended to select higher sparsity regularization parameters, because corrected overlap (due to its correction term) favors highly overlapping, but not dense models. This might explain the reason why the models using the criteria Acc/OC are very sparse (Figure 5). Although a sparse solution seems the most stable for the considered data set, the sparsity of the learned solution is likely to be dataset dependent. Different fMRI datasets might have different levels of sparsity depending on the cognitive task. If a cognitive task only engages a very small network of few regions, then very sparse solutions will probably show the best performance in terms of accuracy and stability. On the other hand, if a cognitive task engages a large network, then less sparse solutions will probably lead to best overall performance. The stability or reproducibility of the model is also an important aspect to be considered for the interpretation as sparsity in itself can produce highly unstable models. Figure 6 illustrates the impact of including reproducibility as a second criterion for model selection for STV. The STV solutions for two different cross validations folds of the LOSO are extremely different when accuracy is used for model selection and become much more similar/stable when correlation or OC is included as a second criterion for model selection.

If we compare the clusters' location for different sparse methods it is interesting to observe that the solutions of LASSO, ENET, STV, and SLAP include basically the same top five regions (with some differences in the ranking order) when accuracy and OC are used as model selection criteria. The top clusters include left middle occipital gyrus (BA 37), right middle occipital gyrus (BA19), left culmen, right middle frontal gyrus (BA 11/10), and right anterior cingulate (BA 24). The fist two regions are known to be involved in visual processing (e.g., Wandell et al., 2007) and the last two have been associated with emotional processing (e.g., Etkin et. al., 2011). The involvement of these regions would be expected in the problem considered, i.e., decoding visualization of emotional pictures.

The potential gains of unstructured vs. structured sparse models for neuroimaging applications will depend on how well the model assumptions (or sparsity penalty) agree with the data structure. Structured sparse models can incorporate more prior knowledge about the data structure and therefore can potentially lead to models with higher performance. However, since neuroimage data, in general, has very high dimensionality and complex structure it is not certain that structured sparse models will have the highest performance when compared with other types of sparsity, as was the case in the present work. Among the penalties considered in the present work, the SLAP penalty seems closer to our beliefs about how the brain works, i.e., the brain is organized in regions and the activities within these regions are expected to vary smoothly. Nevertheless, ENET presented the best performance in terms of accuracy and reproducibility. Our results show that SLAP can lead to very noisy maps with hundreds of cluster, many of them being very small and more likely to be related to noise than brain activity. One difficulty to choose the optimal penalty is the lack of an absolute ground truth in terms of informative or predictive regions in the brain, making it difficult to define an objective criterion for model comparison. Some studies have used results from mass univariate statistical tests between the classes (e.g., *t*-test) as a proxy for the ground truth, however such tests would fail to capture multivariate properties (e.g., subtle differences observed when a set of voxels is considered jointly). Here we investigate the use of two criteria for model selection, decoding accuracy and stability/reproducibility (measured by OC or correlation across LOSO folds). Although these criteria do not embed a metric of distance to the ground truth solution, the combination of decoding accuracy and overlap between the solutions leads to similar solutions across different learning methods.

Rasmussen et al. (2012) have previously investigated the impact of choices of model regularization parameters on the generalization and the stability/reproducibility of spatial patterns extracted from classification models in neuroimaging. The authors evaluate the models using the NPAIRS resampling scheme (i.e., half-splits resamples Strother et al., 2002) and constructed performance-vs.-reproducibility curves (pr-curves) for three classifiers: Support Vector Machine, Fisher Discriminant Analyses and Logistic Regression. For each classifier type, they compared the models that optimized the prediction accuracy, joint prediction accuracy and reproducibility (measured by Pearson correlation coefficient between the models' coefficients), and only reproducibility. The authors observed a trade-off between prediction accuracy and reproducibility and argued that regularization parameters must be selected to balance this trade-off in order to provide a more accurate representation of the underlying brain networks. They also investigated how performance and stability/reproducibility (measured by overlap and mutual information between the solution) varied for the logistic regression with ENET penalty as function of the regularization parameters, however in this case the stability/reproducibility metrics were only used to access the models and not for parameter optimization. Other studies also reported a tradeoff between prediction vs. reproducibility using a penalized Fishers discriminant analysis (FDA) on PCA basis (Strother et al., 2004; Yourganov et al., 2011).

Our work builds upon Rasmussen et al. (2012), as we also investigate the trade-off between prediction accuracy and reproducibility as model selection criteria, but differs from it in many aspects. First, our goal was to investigate the role of model selection criteria on several different sparse methods (LASSO, ENET, TV, LAP, STV, and SLAP). Second, we used a LOSO framework with nested cross-validation for parameter optimization instead of a half-split framework. Third, we investigated two stability/reproducibility metrics, the Pearson correlation coefficient and the pairwise corrected overlap across the LOSO solutions. Using our framework, we observed that when using prediction accuracy and corrected overlap as joint optimization criterion the solutions for different sparse methods become very similar in terms of performance and brain regions identified as relevant. These results suggest that the choice of model regularization parameters might be more important than the choice of the sparsity constraint.

One limitation of our work is to use the LOSO cross-validation framework, i.e., the LOSO framework is known to have high variance and the solutions for different cross-validation folds are not independent. Nevertheless, considering the sample size of 16 subjects, it would be difficult to explore other cross-validation frameworks. It should be noted that since each subject has 42 scans of each active condition, leaving one subject out corresponds to leaving 84 examples out for test. Future work, using larger sample sizes should be performed to investigate the impact of adding stability/reproducibility as model selection criteria in other cross-validation frameworks. A possible future direction could be to explore multitask learning methods, in which the subjects are treated as distinct classification or regression tasks, which are related by means of a joint regularizer (see, for example, Argyriou et al., 2008; Romera-Paredes et al., 2013, and references therein). Ideas from Bzdok et al. (2015) may also prove valuable in this direction.

## Ethics statement

The study was performed in accordance with the local Ethics Committee of the University of North Carolina where the data was originally acquired. Participants had given written informed consent to participate in the study after the study was explained to them.

## Author contributions

LB contributed to designing the experiments, implementing and running the models and writing the paper. JMM and MP contributed to designing the experiments, interpreting the results and writing the paper.

## Funding

JMM was supported by the Wellcome Trust under grant no. WT102845/Z/13/Z. MP was partially supported by EPSRC grants EP/P009069/1 and EP/M006093/1.

### Conflict of interest statement

The authors declare that the research was conducted in the absence of any commercial or financial relationships that could be construed as a potential conflict of interest.

## Footnotes

^2^We remark that there is a typo in the definition of *E* in Baldassarre et al. (2012b), which should coincide with Equation (2) hereafter.

^3^Specifically, Rasmussen et al. (2012) has not employed the accuracy—reproducibility diagram as model selection criterion for sparse models. They have only used it to assess the ENET performance. Whereas in this paper these measures are used both for model selection and assessment of different sparse methods.

^4^Wellcome Department of Imaging Neuroscience, http://www.fil.ion.ucl.ac.uk/spm/.

## References

[B1] Argyriou A. Evgeniou T. Pontil M. (2008). Convex multi-task feature learning. J. Mach. Learn. 73, 243–272. 10.1007/s10994-007-5040-8

[B2] Bach F. Jenatton R. Mairal J. Obozinski G. (2011). Structured Sparsity through Convex Optimization. Technical Report. *Arxiv preprint:1109.2397*.

[B3] Baldassarre L. Morales J. M. Argyriou A. Pontil M. (2012a). A general framework for structured sparsity via proximal optimization, in International Conference on Artificial Intelligence and Statistics (La Palma), 82–90.

[B4] Baldassarre L. Mourao-Miranda J. Pontil M. (2012b). Structured sparsity models for brain decoding from fMRI data, in International Workshop on Pattern Recognition in NeuroImaging (London), 5–8. 10.1109/prni.2012.31

[B5] Belilovsky E. Argyriou A. Varoquaux G. Blaschko M. (2015). Convex relaxations of penalties for sparse correlated variables with bounded total variation. Mach. Learn. 100, 533–553. 10.1007/s10994-015-5511-2

[B6] Bzdok D. Eickenberg M. Grisel O. Thirion B. Varoquaux G. (2015). Semi-supervised factored logistic regression for high-dimensional neuroimaging data. Adv. Neural Inform. Process. Syst. 28, 3348–3356. Available online at: http://papers.nips.cc/paper/5646-semi-supervised-factored-logistic-regression-for-high-dimensional-neuroimaging-data.pdf

[B7] Chambolle A. (2004). An algorithm for total variation minimization and applications. J. Math. Imaging Vis. 20, 89–97. 10.1023/B:JMIV.0000011321.19549.88

[B8] Cortes C. Vapnik V. (1995). Support vector networks. Mach. Learn. 20, 273–297. 10.1007/BF00994018

[B9] Dohmatob E. Gramfort A. Thirion B. Varoquaux G. (2014). Benchmarking solvers for TV-l1 least-squares and logistic regression in brain imaging, in International Workshop on Pattern Recognition in Neuroimaging (PRNI) (Tübingen: IEEE), 1–4.

[B10] Eickenberg M. Dohmatob E. Thirion B. Varoquaux G. (2015). Grouping total variation and sparsity: statistical learning with segmenting penalties, in Medical Image Computing and Computer-Assisted Intervention – MICCAI 2015: 18th International Conference, October 5-9, Proceedings, Part I (Munich), 685–693.

[B11] Etkin A. (2011). Emotional processing in anterior cingulate and medial prefrontal. Trends Cogn. Sci. 15, 85–93. 10.1016/j.tics.2010.11.004 21167765 PMC3035157

[B12] Fiot J. B. Raguet H. Risser L. Cohen L. D. Fripp J. Vialard F. X. (2014). Longitudinal deformation models, spatial regularizations and learning strategies to quantify alzheimer's disease progression. Neuroimage 4, 718–729. 10.1016/j.nicl.2014.02.002 24936423 PMC4053641

[B13] Gramfort A. Thirion B. Varoquaux G. (2013). Identifying predictive regions from fMRI with TV-l1 prior, in International Workshop on Pattern Recognition in Neuroimaging (PRNI) (Philadelphia, PA), 17–20. 10.1109/prni.2013.14

[B14] Grosenick L. Klingenberg B. Katovich K. Knutson B. Taylor J. (2013). Interpretable whole-brain prediction analysis with graphnet. Neuroimage 72, 304–321. 10.1016/j.neuroimage.2012.12.062 23298747

[B15] Grosenick L. Klingenberg B. Katovich K. Knutson B. Taylor J. E. (2011). A family of interpretable multivariate models for regression and classification of whole-brain fMRI data. ArXiv e-prints 1110.4139.

[B16] Hardoon D. Mourao-Miranda J. Brammer M. Shawe-Taylor J. (2007). Unsupervised analysis of fMRI data using kernel canonical correlation. Neuroimage 37, 1250–1259. 10.1016/j.neuroimage.2007.06.017 17686634

[B17] Haynes J. (2015). A primer on pattern-based approaches to fMRI: principles, pitfalls, and perspectives. Neuron 85, 257–270. 10.1016/j.neuron.2015.05.025 26182413

[B18] Haynes J. Sakai K. Rees G. Gilbert S. Frith C. Passingham R. E. (2007). Reading hidden intentions in the human brain. Curr. Biol. 17, 323–328. 10.1016/j.cub.2006.11.072 17291759

[B19] Hoyos-Idrobo A. Schwartz Y. Varoquaux G. Thirion B. (2015). Improving sparse recovery on structured images with bagged clustering, in International Workshop on Pattern Recognition In Neuroimaging (PRNI) (Palo Alto, CA), 73–76.

[B20] Jenatton R. Gramfort A. Michel V. Obozinski G. Eger E. Bach F. (2011). Multi-scale mining of fMRI data with hierarchical structured sparsity. ArXiv e-prints 1105.0363.

[B21] Jenatton R. Gramfort A. Michel V. Obozinski G. Eger E. Bach F. (2012). Multiscale mining of fMRI data with hierarchical structured sparsity. SIAM J. Imaging Sci. 5, 835–856. 10.1137/110832380

[B22] Kloppel S. Abdulkadir A. Jack C. R. J. Koutsouleris N. Mourao-Miranda J. Vemur J. (2012). Diagnostic neuroimaging across diseases. Neuroimage 61, 457–463. 10.1016/j.neuroimage.2011.11.002 22094642 PMC3420067

[B23] Ma S. Yin W. Zhang Y. Chakraborty A. (2008). An efficient algorithm for compressed MR imaging using total variation and wavelets, in Computer Vision and Pattern Recognition, 2008. CVPR 2008. IEEE Conference on (IEEE) (Anchorage, AK), 1–8.

[B24] Micchelli C. A. Morales J. M. Pontil M. (2013). Regularizers for structured sparsity. Adv. Comput. Math. 38, 455–489. 10.1007/s10444-011-9245-9

[B25] Michel V. Gramfort A. Varoquaux G. Eger E. Thirion B. (2011). Total variation regularization for fMRI-based prediction of behavior. IEEE Trans. Med. Imaging 30, 1328–1340. 10.1109/TMI.2011.2113378 21317080 PMC3336110

[B26] Mohr H. Wolfensteller U. Frimmel S. Ruge H. (2015). Sparse regularization techniques provide novel insights into outcome integration processes. Neuroimage 104, 163–176. 10.1016/j.neuroimage.2014.10.025 25467302

[B27] Mourao-Miranda J. Friston K. Brammer M. (2007). Dynamic discrimination analysis: a spatial-temporal svm. Neuroimage 36, 88–99. 10.1016/j.neuroimage.2007.02.020 17400479

[B28] Mourao-Miranda J. Reynaud E. McGlone F. Calvert G. Brammer M. (2006). The impact of temporal compression and space selection on svm analysis of single-subject and multi-subject fMRI data. Neuroimage 33, 1055–1065. 10.1016/j.neuroimage.2006.08.016 17010645

[B29] Pereira F. Mitchell T. Botvinick M. (2009). Machine learning classifiers and fMRI: a tutorial overview. Neuroimage 45, S199–S209. 10.1016/j.neuroimage.2008.11.007 19070668 PMC2892746

[B30] Polyn S. M. Natu V. S. Cohen J. D. Norman K. A. (2005). Category-specific cortical activity precedes retrieval during memory search. Science 310, 1963–1966. 10.1126/science.1117645 16373577

[B31] Rasmussen P. M. Hansen L. K. Madsen K. H. Churchill N. W. Strother S. C. (2012). Model sparsity and brain pattern interpretation of classification models in neuroimaging. Patt. Recogn. 45, 2085–2100. 10.1016/j.patcog.2011.09.011

[B32] Romera-Paredes B. Aung M. H. Bianchi-Berthouze N. Pontil M. (2013). Multilinear multitask learning, in Proceedings of the 30th International Conference on Machine Learning (ICML) (Atlanta, GA), 1444–1452.

[B33] Rudin L. Osher S. Fatemi E. (1992). Nonlinear total variation based noise removal algorithms. Physica D 60, 259–268. 10.1016/0167-2789(92)90242-F

[B34] Schrouff J. Kusse C. Wehenkel L. Maquet P. Phillips C. (2012). ecoding semi-constrained brain activity from fMRI using support vector machines and gaussian processes. PLoS ONE 7:e35860. 10.1371/journal.pone.0035860 22563410 PMC3338538

[B35] Strother S. Anderson J. Hansen L. Kjems U. Kustra R. Sidtis J.. (2002). The quantitative evaluation of functional neuroimaging experiments: the npairs data analysis framework. Neuroimage 15, 747–771. 10.1006/nimg.2001.1034 11906218

[B36] Strother S. Conte S. L. Hansen L. K. Anderson J. Zhang J. Pulapura S.. (2004). Optimizing the fMRI data-processing pipeline using prediction and reproducibility performance metrics: I. A preliminary group analysis. Neuroimage 23(Suppl. 1), S196–S207. 10.1016/j.neuroimage.2004.07.022 15501090

[B37] Talairach P. Tournoux J. (1988). A Stereotactic Coplanar Atlas of the Human Brain. Stuttgart: Thieme.

[B38] Tibshirani R. (1996). Regression shrinkage and selection via the lasso. J. R. Stat. Soc. Ser. B 58, 267–288.

[B39] Tikhonov A. N. Arsenin V. Y. (1977). Solutions of Ill-Posed Problems. Washington, DC: John Wiley.

[B40] Wandell B. A. Dumoulin S. O. Brewer A. A. (2007). Visual field maps in human cortex. Neuron 56, 366–383. 10.1016/j.neuron.2007.10.012 17964252

[B41] Wang Y. Zheng J. Zhang S. Duan X. Chen H. (2014). Randomized structural sparsity via constrained block subsampling for improved sensitivity of discriminative voxel identification. *ArXiv e-prints 1410.4650*. 26027884 10.1016/j.neuroimage.2015.05.057

[B42] Yourganov G. Chen X. Lukic A. S. Grady C. L. Small S. L. Wernick M. N.. (2011). Dimensionality estimation for optimal detection of functional networks in bold fMRI data. Neuroimage 56, 531–543. 10.1016/j.neuroimage.2010.09.034 20858546 PMC3052418

[B43] Zou H. Hastie T. (2005). Regularization and variable selection via the elastic net. J. R. Stat. Soc. Ser. B (Stat. Methodol.) 67, 301–320. 10.1111/j.1467-9868.2005.00503.x

