# Supplementary material for: Sparsity Is Better with Stability: Combining Accuracy and Stability for Model Selection in Brain Decoding
Source: Front Neurosci. 2017 Feb 17;11:62. doi: 10.3389/fnins.2017.00062 (PMC5313509; doi:10.3389/fnins.2017.00062)
Supplement: Supplementary file 1 [file DataSheet1.pdf]

Sparsity is better with stability: combining accuracy and stability for model selection in brain decoding - Supplementary material

Luca Baldassarre, Massimiliano Pontil and Janaina Mourao-Miranda

Appendix

A Optimisation

Given the high-dimensionality of the problem ($p \approx 10^5$ in the following experiments), we use first-order proximal optimisation methods to solve the optimisation problem

$$\min_{\beta \in R^p} \Big\{ E(\beta) + \Omega(\beta) \Big\}. \tag{1}$$

Specifically, we employ FISTA (Beck and Teboulle, 2009), an accelerated proximal-gradient method, that is a form of gradient-descent that can deal with non-smooth functions and scales nicely to large problem sizes. This iterative method can minimise objective functions that are composed by the sum of a smooth term, f, and a non-smooth term g. The method consists of three basic steps: i) computation of the gradient of the smooth term; ii) computation of the proximity operator of the non-smooth term, and iii) an accelerated step which updates the current estimate of the solution as a function of two previous estimates. The pseudo-code for FISTA is reported in Algorithm 1. The proximity operator (Moreau, 1962) associated to the convex function $g : \mathbb{R}^p \to \mathbb{R}$ and evaluated at $y \in \mathbb{R}^p$, is defined as

$$\mathrm{prox}_{\lambda g}(y) = \mathrm{argmin} \left\{ \frac{1}{2}\|x - y\|^2 + g(x) : x \in \mathbb{R}^p \right\}. \tag{2}$$

FISTA requires ∇f to be Lipschitz continuous, that is, there must exist a constant $L > 0$ such that, for all $x, x' \in \mathbb{R}^p$, it holds

$$\|\nabla f(x) - \nabla f(x)\| \leq L\|x - x'\| \ .$$

Algorithm 1 Accelerated Proximal-Gradient Method

$x_1, \alpha_1 \leftarrow 0, \theta_1 \leftarrow 1$
while not converged **do**
  $x_{t+1} \leftarrow \text{prox}_{\frac{g}{L}}\left(\alpha_t - \frac{1}{L}\nabla f(\alpha_t)\right)$.
  $\theta_{t+1} \leftarrow \frac{1+\sqrt{1+4\theta_t^2}}{2}$.
  $\alpha_{t+1} \leftarrow x_{t+1} + \frac{\theta_t - 1}{\theta_{t+1}}(x_{t+1} - x_t)$.
end while

We stop FISTA, when the relative decrease in objective function between two iterates is below 10^{-3} or we have reached 10^4 iterations. When computing the solutions for different regularisation parameter values, we start by solving the problem with the highest value and using the 'warm restart' strategy. That is, we use as starting solution β_0 for the next problem, the final solution obtained for the previous value of the regularisation parameter and so on, until we solve for the smallest regularisation parameter value.

For the GraphNet method, we have $f(\beta) = E(\beta) + \frac{1}{2}\lambda(1-\alpha)\sum_{i\sim j}(\beta_i - \beta_j)^2$ and $L = \frac{\|XX^\top\|}{m} + \lambda(1-\alpha)\|G\|$, where $\|\cdot\|$ is the spectral norm and G is the graph Laplacian of the adjacency graph connecting each voxel to its neighbours. For all the other methods, $f(\beta) = E(\beta)$ and $L = \frac{\|XX^\top\|}{m}$.

For the Lasso and the GraphNet methods, we have that $g(\beta) = \lambda_*\|\beta\|_1$, with $\lambda_* = \lambda_1$ for the LASSO and $\lambda_* = \lambda\alpha$ for the GraphNet and

$$\text{prox}_{\frac{g}{L}}(y) = \left(|y| - \frac{\lambda_*}{L}\right)_+ \text{sign}(y)$$

where $(y)_+ = y$ if $y > 0$ and zero otherwise and both $(\cdot)_+$ and *sign* are applied component-wise.

For the methods that employ the Total Variation penalty, we compute the proximity operator numerically. For this purpose, we rewrite the regulariser as a composite function

$$\Omega(\beta) = \|B\beta\|_1 \ ,$$

where $B = \nabla$, the discrete gradient operator in 3 dimensions, for the Total

Variation method, and $B = \begin{bmatrix} \nabla \\ I \end{bmatrix}$ for the Sparse Total Variation method (I is the $p \times p$ identity matrix). We use the fixed-point scheme in Argyriou et al. (2011) and Micchelli et al. (2011) to compute the proximity operator of composite functions $g \circ B$, in which prox_g has a closed form expression.

References

Argyriou, A., Micchelli, C., Pontil, M., Shen, L., Xu, Y., 2011. Efficient first order methods for linear composite regularizers. Arxiv preprint:1104.1436 .

Beck, A., Teboulle, M., 2009. A fast iterative shrinkage-thresholding algorithm for linear inverse problems. SIAM Journal on Imaging Sciences 2, 183–202.

Micchelli, C., Shen, L., Xu, Y., 2011. Proximity algorithms for image models: denoising. Inverse Problems 27, 045009.

Moreau, J., 1962. Fonctions convexes duales et points proximaux dans un espace hilbertien. CR Acad. Sci. Paris Sér. A Math 255, 2897–2899.
